# The Prevalence of Diagnosed Hypertension and Its Determinants in Zakho City, Kurdistan Region, Iraq

**DOI:** 10.7759/cureus.81989

**Published:** 2025-04-10

**Authors:** Nawfal R Hussein, Ali Abdi, Ibrahim A Naqid, Delovan S Mahfodh, Brisik Rashad

**Affiliations:** 1 Department of Biomedical Sciences, College of Medicine, University of Zakho, Zakho, IRQ; 2 Department of Medicine, College of Medicine, University of Duhok, Duhok, IRQ

**Keywords:** cardiovascular disease, hypertension, prevalence, public health, zakho

## Abstract

Introduction and aims: Hypertension (HTN) is a chronic and common condition that causes health issues, such as stroke and heart failure, causing substantial disease burden and mortality. This study aimed to explore the prevalence of HTN and determine its risk factors in Zakho, Iraq.

Methodology: This cross-sectional study occurred between 15 January 2024 and 18 March 2024. A multi-stage sampling method was utilized, with interviewers administering questionnaires to random households selected by a number generator.

Results: A total of 537 participants were included, comprising 64.2% (n = 345) males and 35.8% (n = 192) females, with an average age of 35.75 (±13.89). The study showed that the prevalence of HTN was 9.5% (n = 51). A univariate analysis revealed that increasing age (p < 0.001), high waist circumference (p < 0.001), no education (p < 0.001), marriage (p = 0.001), unemployment (p = 0.04), non-smoking (p = 0.03), hyperlipidemia (p < 0.001), and ischemic heart disease (p = 0.003) were significantly associated with HTN. The multivariate logistic regression revealed that hyperlipidemia (adjusted odds ratio (aOR) = 3.729; 95% CI: 1.762-7.891), age (aOR = 1.056; 95% CI: 1.030-1.083), and non-smoking (aOR = 0.333; 95% CI: 0.142-0.781) were significant predictors of HTN, with p-values of 0.001, <0.001, and 0.011, respectively.

Conclusions: A high prevalence of HTN was found among those having multiple comorbidities and was significantly associated with age and hyperlipidemia. Public awareness campaigns, targeted intervention for high-risk groups, and healthier lifestyles are crucial to combat HTN.

## Introduction

Hypertension (HTN) is a prevalent chronic condition that is becoming increasingly epidemic on a global scale, paralleling the rising rates of obesity, metabolic syndrome, and type 2 diabetes [1]. Factors contributing to HTN include poor dietary habits, lack of physical activity, tobacco and alcohol use, obesity, genetic predisposition, age above 65, and exposure to environmental toxins [2]. It is widely recognized that poorly managed HTN can lead to severe health issues, such as stroke and heart failure, which rank among the top causes of death globally [3]. The World Health Organization (WHO) reports that approximately one billion adults are affected by HTN, resulting in over nine million deaths each year [4]. Research has shown that managing high blood pressure effectively can greatly reduce the risk of cardiovascular problems and mortality [5]. There is a strong association of HTN with a wide range of chronic diseases, including metabolic syndrome and obesity, cardiovascular disease, and stroke [6,7]. The worldwide incidence and impact of HTN have risen over the last 10 years, attributed to an aging demographic, shifts in lifestyle and behavioral risks, and insufficient physical activity, particularly in developing nations [1]. Additionally, the global levels of awareness, treatment, and management of HTN remain notably low, with significant variations observed among various countries and socioeconomic categories [1]. In 2006, a study conducted in Iraq on risk factors for chronic noncommunicable diseases revealed that the prevalence of HTN was 40.4% [8]. In 2008, the WHO released health data for the Eastern Mediterranean Region, showing that the rate of HTN in Iraq for both sexes was 29.4% (varying from 20.4% to 38.9%) [8]. A household survey conducted in Thi-Qar Governorate in 2014 revealed that the total prevalence of HTN was 26.5% [9]. Prevalence data on HTN in our region are sparse. Therefore, the main aim of this paper is to fill this gap by providing a comprehensive analysis of the prevalence of diagnosed HTN and its related risk factors in Zakho City, Kurdistan Region, Iraq.

## Materials and methods

Study design

A community-based cross-sectional survey was carried out between 15 January 2024 and 18 March 2024 in Zakho City, located in the Kurdistan Region of Iraq. The focus of the study was on residents aged 18 years and older. A multistage sampling method was utilized. Within the selected areas, households were randomly selected using a random number generator. Since the actual population proportion was unknown, a proportion (p) of 0.5 was assumed to guarantee the maximum sample size. The initial sample size was determined using the formula for an infinite population, resulting in a final sample size of around 384. In total, 537 individuals participated in the study (345 males and 192 females), with a mean age of 35.75 years and a standard deviation of 13.89 years.

Inclusion and exclusion criteria

The study included individuals aged 18 years or older who were residing in Zakho City. Those younger than 18 years of age were excluded to ensure compliance with legal and ethical research standards. Additionally, individuals who chose not to participate, whether due to personal preference or other reasons, were excluded from the study. All other individuals were deemed eligible, including known cases of HTN and other chronic diseases.

Data collection

Data were gathered through a pretested structured questionnaire administered by an interviewer. The questionnaire included information on sociodemographic, behavioral, and clinical attributes. Weight was assessed using a high-quality weighing scale, and height was recorded while standing. Body mass index (BMI) was computed by dividing weight in kilograms by height in meters squared. A BMI of less than 25 kg/m² was deemed normal, 25-29.9 kg/m² was classified as overweight, and 30 kg/m² or greater was categorized as obese, with 17 participants with morbid obesity (BMI>40) [10]. Waist circumference was determined by positioning a tape measure around the body at the point between the lowest rib margin and the top of the iliac crest. A waist circumference of 94 cm or less in men and 80 cm or less in women was considered normal. A waist circumference more than 94 cm in men and 80 cm in women was classified as high. Physical activity encompasses any movement of the body produced by skeletal muscles that necessitates energy use [10]. To identify chronic diseases, including hypertension, participants were asked to provide information regarding their official government-issued identification for chronic disease status.

Statistical analysis

The information was encoded, cleaned, inputted, and updated using Microsoft Excel (Microsoft Corporation, Redmond, WA) and subsequently exported to IBM SPSS Statistics for Windows (version 26.0; IBM Corp., Armonk, NY) for additional analysis. To describe the study cohort, descriptive statistics were calculated and displayed in tables and narrative form, incorporating means, medians, percentages, and standard deviations. The chi-squared test and univariate and multivariate analyses were used to analyze factors associated with overweight and obesity. A p-value of less than 0.05 was deemed to indicate statistical significance for a variable.

Ethics

Ethical approval was granted by the Scientific Research and Ethics Committee at the College of Medicine, University of Zakho (approval number: OCT2023/UOZE42). The study procedure, design, and its aims were explained to the participants. Prior to administering the questionnaire, each respondent provided verbal informed consent.

## Results

The study included 537 participants, their ages spanning a wide range, but on average, they were around 35.75 years old (±13.89). Men outnumbered women, with 345 (64.2%) males compared to 192 (35.8%) females. Additionally, 324 (60.3%) participants were married. Regarding education, 365 (68.0%) were educated, and 285 (53.1%) were employed. Smoking was widespread, with 357 (66.5%) participants identifying as smokers. Physical activity, on the other hand, was less common - 319 (59.4%) participants described themselves as inactive. When asked about sleep, 341 (63.5%) reported having no disturbances (Table 1).

**Table 1:** Sociodemographic and behavioral characteristics of the study participants (n = 537) SD: standard deviation

Variable		Number (N)	Percentage (%)	
Age				
Mean ± SD		35.75 ± 13.89		
Sex				
Male		345	64.2	
Female		192	35.8	
Education				
Uneducated	172			32.0
Educated	365			68.0
Marital status				
Single	213			39.7
Married	324			60.3
Employment				
Unemployed	252			46.9
Employed	285			53.1
Smoking status				
Non-smoker	357			66.5
Smoker	180			33.5
Physical activity				
Not physically active	319			59.4
Physically active	218			40.6
Sleep disturbance				
No	341			63.5
Yes	196			36.5

With regard to clinical characteristics, 51 (9.5%) participants had HTN, 75 (14.0%) had a history of hyperlipidemia, and 20 (3.7%) had a history of ischemic heart disease (Table 2). Only 16 (3.0%) had a thyroid disease history, and 18 (3.4%) had a history of diabetes. The average ± SD of sleep time was 6.88 ± 1.86. The average ± SD of waist circumference was 90.84 ± 15, and of BMI was 27.88 ± 10.09 (Table 2).

**Table 2:** Clinical characteristics of the study participants (n = 537) SD: standard deviation; DM: diabetes mellitus; BMI: body mass index

Variable		Number (N)	Percentage (%) / Mean ± SD	
Hypertension				
No	486			90.5
Yes	51			9.5
Hyperlipidemia				
No	462			86
Yes	75			14.0
Ischemic heart disease				
No	517			96.3
Yes	20			3.7
Thyroid disease				
No	521			97.0
Yes	16			3.0
DM status				
No	519			96.6
Yes	18			3.4
Activity time	-			7.69 ± 6.39
Sleep duration	-			6.88 ± 1.86
Waist circumference	-			90.84 ± 15
BMI	-			27.88 ± 10.09

Prevalence of HTN

The prevalence rate in the study sample was 9.5% (n = 51) (95% CI: 7.0-12.0), and 10.4% (n = 20) of females had HTN, slightly higher than the 9.0% (n = 31) of males (p = 0.588). Higher prevalence rates were seen in certain sociodemographic groups, such as the married (13.3%; n = 43; p = 0.01), uneducated (18.0%; n = 31; p < 0.01), and unemployed (12.3%; n = 31; p = 0.04) groups. Interestingly, 11.5% (n = 41) of non-smokers were hypertensive, higher than the 5.6% (n = 10) of smokers (p = 0.03). Slightly higher rates (10.1%; n = 22) were seen in physically active participants compared to physically inactive participants (9.1%; n = 29; p = 0.698). Almost equal rates were observed between groups of sleep disturbance status (9.7% (n = 33) without sleep disturbance versus 9.2% (n = 18) with sleep disturbance) (p = 0.851) (Table 3).

**Table 3:** Factors associated with hypertension: univariate and multivariate analyses (n = 537) SD: standard deviation; COR: crude odds ratio; aOR: adjusted odds ratio; DM: diabetes mellitus; BMI: body mass index

Variables	Non-hypertensive N (%)	Hypertensive N (%)	Univariate analysis			Multivariate analysis	
			P-value		COR (95% CI)	P-value	aOR (95% CI)
Sociodemographic variables							
Sex							
Male	314 (91.0)	31 (9.0)	0.588	1.178 (0.652, 2.129)		0.717	0.858 (0.376, 1.961)
Female	172 (89.6)	20 (10.4)					
Education							
Uneducated	141 (82.0)	31 (18.0)	<0.001	0.264 (0.145, 0.478)		0.289	0.653 (0.297, 1.435)
Educated	345 (94.5)	20 (5.5)					
Marital status							
Single	205 (96.2)	8 (3.8)	0.001	3.921 (1.805, 8.519)		0.289	1.719 (0.631, 4.684)
Married	281 (86.7)	43 (13.3)					
Employment							
Unemployed	221 (87.7)	31 (12.3)	0.04	0.538 (0.298, 0.97)		0.057	0.451 (0.198, 1.026)
Employed	265 (93.0)	20 (7.0)					
Smoking status							
Non-smoker	316 (88.5)	41 (11.5)	0.03	0.453 (0.222, 0.928)		0.011	0.333 (0.142, 0.781)
Smoker	170 (94.4)	10 (5.6)					
Physical activity							
No	290 (90.9)	29 (9.1)	0.698	1.122 (0.627, 2.011)		0.143	1.692 (0.836, 3.425)
Yes	196 (89.9)	22 (10.1)					
Sleep disturbance							
No	308 (90.3)	33 (9.7)	0.851	0.944 (0.516, 1.725)		0.280	0.666 (0.319, 1.391)
Yes	178 (90.8)	18 (9.2)					
Clinical variables							
Hyperlipidemia							
No	430 (93.1)	32 (6.9)	<0.001	4.559 (2.423, 8.580)		0.001	3.729 (1.762, 7.891)
Yes	56 (74.7)	19 (25.3)					
Ischemic heart disease							
No	472 (91.3)	45 (8.7)	0.003	4.495 (1.647, 12.27)		0.711	0.783 (0.216, 2.844)
Yes	14 (70.0)	6 (30.0)					
Thyroid disease							
No	472 (90.6)	49 (9.4)	0.679	1.376 (0.304, 6.232)		0.670	0.694 (0.130, 3.710)
Yes	14 (87.5)	2 (12.5)					
DM status							
No	472 (90.9)	47 (9.1)	0.073	2.869 (0.908, 9.070)		0.639	1.39 (0.352, 5.489)
Yes	14 (77.8)	4 (22.2)					
Continuous variables							
Age	34.32 ± 12.951	49.73 ± 14.862	<0.001	1.071 (1.049, 1.092)		<0.001	1.056 (1.030, 1.083)
Sleep duration	6.88 ± 1.85	6.84 ± 2.02	0.897	0.99 (0.848, 1.155)		0.514	1.063 (0.884, 1.279)
Waist circumference	90.22 ± 14.56	98.18 ± 13.79	<0.001	1.038 (1.017, 1.059)		0.469	1.011 (0.982, 1.041)
BMI	27.74 ± 10.43	29.55 ± 5.68	0.241	1.013 (0.992, 1.034)		0.452	0.977 (0.919, 1.038)

Higher rates were observed among hyperlipidemic (25.3%; n = 19; p < 0.001), ischemic heart disease (30.0%; n = 6; p < 0.003), and thyroid disease (12.5%; n = 2; p = 0.679) cases. However, 9.1% (n = 47) of non-diabetic cases were hypertensive (Table 3).

Factors associated with HTN

In univariate analysis, results showed significant associations with increasing age (crude odds ratio (COR) = 1.071; 95% CI: 1.049-1.092; p < 0.001) and waist circumference (COR = 1.038; 95% CI: 1.017-1.059; p < 0.001). More significant associations were also found among uneducated (COR = 0.264; 95% CI: 0.145-0.478; p < 0.001), married (COR = 3.921; 95% CI: 1.805-8.519; p = 0.001), unemployed (COR = 0.538; 95% CI: 0.298-0.97; p = 0.04), and non-smoking participants (COR = 0.453; 95% CI: 0.222-0.928; p = 0.03). Hyperlipidemic (COR = 4.559; 95% CI: 2.423-8.580; p < 0.001) and ischemic heart disease (COR = 4.495; 95% CI: 1.647-12.27; p = 0.003) cases were also shown to be significantly associated.

Multivariate logistic regression results showed significant associations with some variables, such as hyperlipidemia (adjusted odds ratio (aOR) = 3.729; 95% CI: 1.762-7.891; p = 0.001), age (aOR = 1.056; 95% CI: 1.030-1.083; p < 0.001), and smoking status (aOR = 0.333; 95% CI: 0.142-0.781; p = 0.011) (Table 3).

## Discussion

HTN is a consistent and major public health concern due to its increasing prevalence and burden globally, especially in low- and middle-income countries, such as Iraq [11]. In this study, we aimed to investigate the prevalence of diagnosed HTN cases and its associated risk factors in Zakho City. This study is of importance especially in such an area where electronic patient registration systems are not yet implemented.

In this study, the prevalence of diagnosed HTN was 9.5% (n = 51) (95% CI: 7.0-12.0). This is much lower than previous study results reported in Erbil City (54.9%), Duhok Governorate (41.3%), Baghdad (24%), and that reported among Iraqi adults (35.6%) in 2022 [8,12-14]. The prevalence of this study was also lower than that reported in neighboring countries, such as Kuwait (15%), Iran (22%), Syria (40.5%), Turkey (44%), Jordan (32.3%), and the overall pooled prevalence in the Middle East (29.5%) [15-20]. The main difference between our research and earlier studies that report a higher incidence of HTN is in the diagnostic methodology employed. Our research was based exclusively on the medical histories of participants, considering only those who had a confirmed and previously identified diagnosis of HTN. In contrast, other studies calculated the prevalence of HTN by measuring participants' blood pressure directly during the study, which likely identified cases that were undiagnosed as well [8]. This difference in methodology may account for the lower prevalence found in our research, as it omits individuals with undiagnosed or asymptomatic HTN who could have been detected through direct blood pressure assessments. Furthermore, a study among Kurds in the Ravansar district, Iran, reported an overall prevalence of 15.7%, higher than the present study [21]. We found that the prevalence rates of our participants aged 35-45 and 46-55 were 8.6% (n = 12) and 22.7% (n = 15), respectively, which were higher than the rates of corresponding age groups in the Ravansar study, 5.3% and 16.3%, respectively.

We found a significant, independent association between HTN and increasing age (p-value < 0.001), which is consistent with previous regional and global findings [8,11,12,14,22]. There are multiple mechanisms for this, including mechanical hemodynamic changes, arterial stiffness, neurohormonal and autonomic dysregulation, and the aging kidney [23]. Hyperlipidemia was another significant and independent variable associated with HTN, and although this variable was not explored in most of the aforementioned studies, dyslipidemia is known to be highly prevalent among hypertensive patients in Middle Eastern countries, such as Lebanon, Egypt, and Saudi Arabia [24-26]. The mechanisms for this could be several, as dyslipidemia causes endothelial dysfunction, which disrupts nitric oxide production and BP regulation [27,28], causes the loss of physiological vasomotor activity [29], and decreases the distensibility of large elastic arteries [30]. The prevalence of diabetes in our study is very low; further studies are required to confirm this finding.

Interestingly, our study found a significant paradoxical association between non-smokers and HTN (aOR = 0.333). This contrasts with regional studies done in the Duhok governorate and the Middle East [12,20]. While some studies found no significant associations between smoking status and HTN, a study from Turkey found an association between non-smokers and ex-smokers with HTN, similar to our results [18]. It is important to note that we do not refute smoking as a main risk factor for HTN and that there can be other explanations for this paradoxical finding. First, two-thirds of our participants were non-smokers (66.5%; n = 357), which may have reduced the statistical power needed to detect associations in the smoker group. Second, smoking was self-reported and prone to bias, and this may have contributed to our finding since self-reported accuracy of smoking is known to be underestimated internationally [31]. Finally, despite adjusting for other variables in multivariate analysis, residual confounding factors may have contributed to this observed association.

This study was community-based and involved an appropriate sample size in a city where similar studies on HTN are lacking, providing insightful new epidemiological data. Our study involved diagnosed HTN cases and compared them with regional data, providing clues into awareness levels in the population. However, this study has several limitations, most notably, reliance on already diagnosed cases of HTN as opposed to measuring BP. The main limitation of the study is that it depends on the history of HTN. Moreover, the cross-sectional design renders it difficult to establish causal relationships and is subject to recall bias regarding self-reported data such as behavioral and clinical characteristics, possibly resulting in an underestimation of the true HTN prevalence.

## Conclusions

Our research indicates a significantly reduced rate of diagnosed HTN in Zakho City when compared to regional and global estimates. This indicates that there may be undiagnosed HTN in our region. Nevertheless, this variance is primarily linked to differences in diagnostic techniques, since our research depended on medical history, instead of direct blood pressure assessment. The results highlight the critical necessity to improve HTN screening, early identification, and awareness initiatives to close the gap between diagnosed and undiagnosed instances. Moreover, the significant link between hypertension, aging, and hyperlipidemia reinforces the necessity of thorough cardiovascular risk management approaches. To combat hypertension, nationwide screening, regular blood pressure checks, and public awareness campaigns are essential. Targeted interventions for high-risk groups, digital health tools, smoking cessation programs, and healthier diets should be prioritized. Ongoing research is crucial to inform future public health policies.

## Appendices

**Table 4:** The questionnaire of the prevalence of diagnosed hypertension and its determinants hrs: hours; ID: identification; DM: diabetes mellitus; IHD: ischemic heart disease

Section	Question/Field	Options/Response
Code	Code	(Fill in)
Demographics	Gender	Male / Female
	Age	(Fill in)
	Residence	Urban / Rural
	Educational status	Educated / Uneducated
	Marital status	Single / Married
	Occupation	Employed / Unemployed
Behavioral and Clinical Characteristics	Smoking status	Smoker / Non-smoker
	Sleep disturbance	Yes / No
	Number of sleeping hours	(Fill in)
	Physical activity	Yes (____ hrs/week) / No
Health Status	Do you have hypertension?	Yes / No
	If yes, confirmed by	Chronic diseases ID / Medical report
	Other related diseases	DM / Hyperlipidemia / IHD / Thyroid disease
Anthropometric Measurements	Weight	(Fill in)
	Height	(Fill in)
	Waist circumference	(Fill in)
